# A Case of *Listeria monocytogenes* ST-219 Meningo-Encephalitis

**DOI:** 10.3390/ijerph16010008

**Published:** 2018-12-20

**Authors:** Giovanni Sotgiu, Narcisa Muresu, Marco Dettori, Erica Mura, Andrea Cossu, Maria Dolores Masia, Paola Murgia, Clementina Cocuzza, Enrico Pietro Luigi De Santis, Christian Scarano, Carlo Spanu, Andrea Piana

**Affiliations:** 1 Department of Medical, Surgical and Experimental Sciences, University of Sassari, 07100 Sassari, Italy; gsotgiu@uniss.it (G.S.); eri.mura@tiscali.it (E.M.); andreacossu@uniss.it (A.C.); mdmasia@uniss.it (M.D.M.); piana@uniss.it (A.P.); 2 Department of Biomedical Sciences, University of Sassari, 07100 Sassari, Italy;; 3 Intensive Unit Care, “SS Annunziata” Hospital, 07100 Sassari, Italy; paolamurgia77@tiscali.it; 4 Department of Medicine and Surgery, University of Milano-Bicocca, 20126 Monza, Italy; cocuzza@unimib.it; 5 Department of Veterinary Medicine, University of Sassari, 07100 Sassari, Italy; desantis@uniss.it (E.P.L.D.S.); scarano@uniss.it (C.S.); cspanu@uniss.it (C.S.)

**Keywords:** listeriosis, ST-219, virulence factors, foodborne infection

## Abstract

Listeriosis is a foodborne disease characterized by high hospitalization and fatality rates, especially in vulnerable groups including elderly subjects, pregnant women, etc. We report on the first case of *Listeria monocytogenes* ST-219 meningo-encephalitis in a woman aged 83 years. An epidemiological and molecular investigation was performed to detect the source of infection and the virulence factors associated with *L. monocytogenes* invasiveness in this patient. All environmental- and clinical-associated isolates were found to belong to serotype 4b and ST-219 as well as possessing *actA, prfA, hlyA*, and *rrn* virulence genes. Antibiotic susceptibility testing also detected resistance to cotrimoxazole, clindamycin, erythromycin, and oxacillin in these isolates. Conventional and molecular surveillance of listeriosis cases, based on the systematic assessment of spatio-temporal trends, virulence genes, and antimicrobial susceptibility testing patterns, are key to preventing and controlling the emergence and spread of *L. monocytogenes* strains, including hypervirulent clones.

## 1. Introduction

Listeriosis, one of the most important foodborne infectious diseases, is caused by *Listeria monocytogenes*, which is a facultatively intracellular Gram-positive microorganism. Listeriosisis a clinical and public health issue, associated with high hospitalization and fatality (i.e., 30%) rates, particularly in vulnerable groups, such as the elderly, pregnant women, newborns, and immunocompromised patients [1]. In the United States, listeriosis shows a low estimated annual incidence rate (1600 infections) in comparison with high hospitalization and mortality rates (1400 and 250 cases, respectively) [2]. In adults aged 65 years or older and pregnant women, the annual incidence rate was 1.3 and 3.0 cases per 100,000 population, respectively [3].

Its epidemiological burden can be significant owing to the broad *L. monocytogenes* environmental distribution. The bacteria’s ability to survive in several stress conditions (e.g., low temperature) favors its growth in refrigerated food, such as cheese, raw milk, vegetables, smoked fish, and ready-to-eat meats [4]. The formation of biofilms can help increase its resistance to stress conditions (e.g., biocides and low and high temperature). The biofilm structure favors antimicrobial and resistance removal owing to its composition based on a matrix of extra-cellular polymeric substances.

Gastrointestinal listeriosis, the most frequent clinical form, can be complicated by sepsis, meningo-encephalitis, spontaneous abortion, or death [5]. Bacterial invasiveness has been partially explained by in vitro and ex vivo studies. Several hypotheses on host, *Listeria* spp., and environmental factors have been evaluated. In particular, *L. monocytogenes* shows virulence factors promoting intracellular survival and replication, escape from immune system, and cell-to-cell spread [6]. Expression of virulence genes can be regulated by the primary regulator Positive Regulatory factor A (PrfA), which activates a gene cluster involved in phagosomal lysis, polymerization of actin filaments, and human invasiveness [7].

Out of 13 *L. monocytogenes* serotypes, the majority (95%) of human cases are caused by serovars 1/2a, 1/2b, 1/2c, and 4b [8]. Virulence factors have been found in strains isolated from human cases but not in animal specimens [9].

The worldwide increased reporting of listeriosis cases has been explained by pathogen- (e.g., virulence factors and antibiotic-resistance mechanisms) and host-related (e.g., elderly susceptible patients, high prevalence of chronic medical conditions) factors. In particular, an increasing incidence trend has been described in European countries in 2011–2014 [10]. However, the higher sensitivity of bacteriological diagnostic methods, following the implementation of rapid molecular tools, has contributed to a higher diagnosis and reporting rate [4].

Recent reports highlighted an increased proportion of antimicrobial resistant strains [11] after the first isolation of multidrug-resistant strains in 1988 [12,13]. Gene similarity conferring partial or missing drug susceptibility between *Listeria* spp., *Enterococcus*, and *Streptococcus*, suggests a horizontal transfer of mobile genetic elements and plasmids [11].

Control and prevention of foodborne cases requires an active systematic monitoring and surveillance system, which has been implemented in several high-income countries. In Italy, regional healthcare systems implemented and scaled-up surveillance and notification programs, aimed to control and prevent outbreak episodes associated with food-processing activities. In Sardinia, Italy, a surveillance program was funded and implemented, named “Strategies of active surveillance and networking of *L. monocytogenes* infections”, promoted by the Sardinian Region [14] to prevent human, animal, and environmental transmission.

Herein, we describe a severe case of *L. monocytogenes* infection in an elderly Italian patient and the following preventative and control interventions, as well as its molecular profile, to better understand the role played by virulence factors in invasive disease.

## 2. Case Presentation

Ethical approval and informed consent for this study was unnecessary according to the Italian legislation concerning the guidelines for the performance of observational studies (G.U. n. 76. 31-3-2008).

In January 2018, an 83-year-old female patient with type II diabetes, hypertension, and overweight, and aphasia and two-day high fever (39.2 °C) was initially admitted to the internal medicine department of the University Hospital of Sassari, Italy. After her physical exam, she was immediately transferred to the Intensive Care Unit of the same hospital with a clinical suspicion of meningitis, confirmed by the magnetic resonance imaging findings. Blood, respiratory, urine, and cerebrospinal fluid (CSF) specimens were collected for chemical and bacteriological (both conventional and molecular) analyses.

High protein (>340 mg/dL), low glucose (33 mg/dL), and pleocytosis (>70% mononuclear cells) were found in CSF. *L. monocytogenes* was diagnosed in blood and CSF cultures using conventional and molecular techniques. In particular, the Vitek-2 System (BioMèrieux, Marcy l’Etoile, France) was used for identification, and antibiotic susceptibility was tested using Kirby-Bauer disc diffusion method following the Clinical and Laboratory Standards Institute (CLSI) guidelines. The extraction of bacterial chromosomal DNA was performed using QIAamp DNA Mini kit from blood and body fluids (QIAGEN cat. n. 51304). Molecular identification was carried out by real-time polymerase chain reaction (RT-PCR; EuSepScreen^®^ Lattanti kit, Eurospital cod. 9144). Drug susceptibility testing showed resistance to cotrimoxazole, clindamycin, erythromycin, and oxacillin (Table 1).

Infectious diseases specialists recommended a 3-week regimen of meropenem (2 g thrice per day) [15,16]. After two days of treatment, fever decreased but severe neurological sequalae occurred (e.g., lack of consciousness, muscular hypotonia, and ipoasthenia). After 44 days of ICU stay, the patient was transferred out to the medicine ward in stable clinical condition and, then was discharged with a rehabilitation program prescribed to achieve a neurological recovery.

Following the standards described in the local protocol, an epidemiological investigation was started to detect any environmental sources of the infection [17]. A total of 25 samples were collected from food stored in (e.g., mortadella and cheese) and surfaces of the fridge located in the patient’s house. All of them were positive for *L. monocytogenes*.

**Figure 1:**
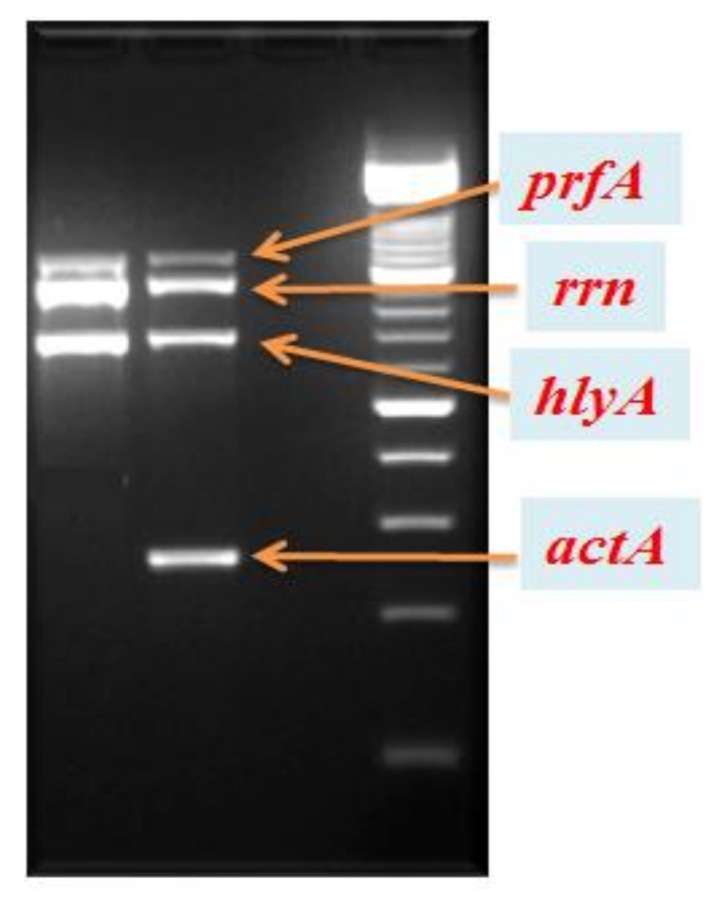
Polymerase chain reaction (PCR)-based virulence genes of *L. monocytogenes* strains isolated from food, environmental surfaces, and biological specimens.

**Figure 2:**
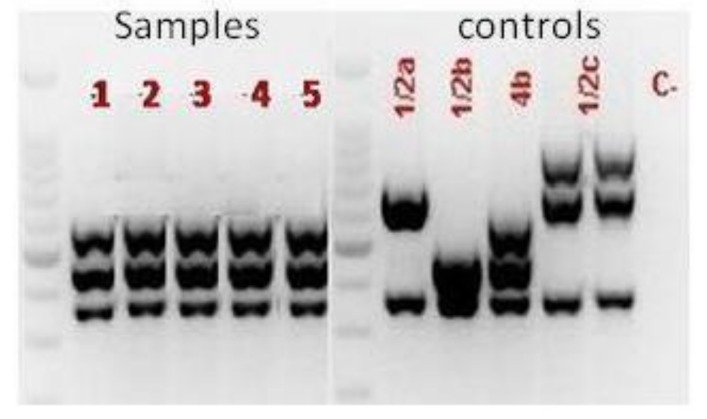
PCR-based serotyping of *L. monocytogenes* strains isolated from food, environmental surfaces, and biological specimens.

Detection of virulence genes (*actA, prfA, hlyA,* and *rrn*) (Figure 1) and serotyping (Figure 2) was performed on all *L. monocytogenes* isolates found in food, environmental surfaces, and biological samples using a multiplex PCR [8,18]. In particular, serotyping was carried out amplifying Imo0737, lmo1118, ORF2819, and ORF2110 sequences, with *prs* gene as positive control. All virulence genes were found in the isolates and the only serotype 4b was diagnosed in all specimens.

**Figure 3:**
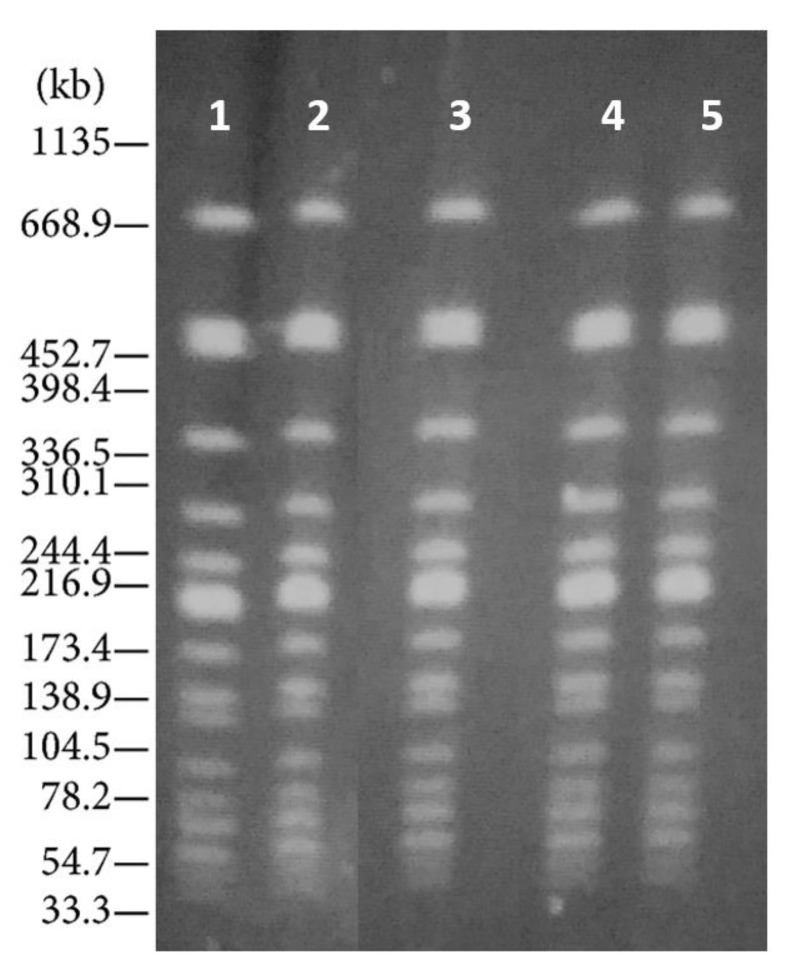
Pulse Field Gel Electrophoresis of *L. monocytogenes* isolates based on the use of the restriction enzyme ApaI. Line 1= Human isolate; Lines 2 and 3= Foodborne isolates; Lines 4 and 5= Environmental isolates. *Salmonella branderup* H9812: Molecular marker.

Isolates were compared using Pulse Field Gel Electrophoresis (PFGE) [19]. They were deemed clonally related if the Dice coefficient was >80%, whereas patterns with indistinguishable PFGE banding patterns (i.e., similarity coefficient >97%) were considered to belong to the same subtype [20]. Only profiles of the same Pulse-type were found (Figure 3).

Multi Locus Sequence Type was completed following the Pasteur Institute protocol [21]. The internal fragments of seven *L. monocytogenes* housekeeping genes (i.e., *gapA, infB, mdh, pgi, phoE, rpoB and tonB*) were sequenced using Bioedit software (Ibis biosciences, Carlsbad, CA) to assess similarity and compatibility. Only the Sequence Type (ST)-219 was found.

## 3. Discussion

Despite considerable advances in food safety and control, listeriosis remains a serious public health issue with high fatality and hospitalization rates in vulnerable population groups [9]. The European Centre for Diseases Control and Prevention estimated 2502 cases in 2017, of which 67% occurred in individuals aged more than 65 years old. About 75% of deaths occurred in the elderly, with a hospitalization rate above 98% [22].

This case report describes the first human case of listeriosis caused by the hypervirulent strain LM-ST-219, which contaminated environmental surfaces and food as proven by the identical molecular profile of the selected isolates.

Previous studies described hyper-and hypo-virulent clones of *L. monocytogenes* mainly associated with human and animal cases, respectively. The dichotomous incidence of serotypes in different beings is associated with a heterogeneous clonal complexity and expression of genes encoding for virulence factors [23].

Hypervirulent clones of *L. monocytogenes* have been shown to exhibit tropism to the central nervous system (CNS) and fetal placenta. ST-219 belongs to Clonal-Complex 4 (CC4), which is usually prevalent in cases of human listeriosis and in non-immunocompromised patients with comorbidities.

Maury et al. [24] reported a cluster of six hypervirulent genes (i.e., *Listeria* pathogenicity Island-4) encoding the putative cellobiose-family associated with CC4 and symptomatic human listeriosis, whereas they were not found in food-associated clones. All isolates we described in our case-report were CC4, i.e., hypervirulent. Detection of virulence factors in our case confirmed a relationship between *L. monocytogenes* invasiveness and *prfA* cluster genes [25].

Four out of 13 serotypes are strongly associated with human listeriosis and are grouped in the Lineage I [26]. Serotype 4b, described in our case report, has been frequently found in human cases and foods, although recent reports showed an increasing incidence of the animal serotype 1/2a and 3a in humans [27]. Specific serotypes are prevalent in some foods and environments; changes in human habits can increase the risk of acquiring serotypes whose incidence has been lowest in the recent past. In particular, several reports highlighted the threat of ready-to-eat foods (e.g., salmon carpaccio, marinated seafood salads, etc.), exposed to mild treatment during their preparation, then at risk of contamination, and frequently purchased by several population groups [28]. The long shelf-life of some products favor the *L. monocytogenes* cross-transmission, as proved in a Finnish 3a serotype-related outbreak linked to the distribution of contaminated butter [29].

Resistance to trimethoprim/sulfamethoxazole (a second-line drug in patients to penicillin) and other antibiotics in our isolates raise the issue of emerging antimicrobial resistance in *L. monocytogenes* strains [11]. The increasing spread of drug-resistant strains and virulence factors associated with specific serotypes may be play a pathogenic role that should be prevented to decrease *L. monocytogenes* fatality and hospitalization rate.

## 4. Conclusions

Our study highlights the importance of safe food handling and storage, particularly when highest risk persons (including elderly people) are exposed to contaminated food. A comprehensive surveillance and control program, as well as etiological distribution of hypervirulent strains in humans and animals, is needed to assess epidemiological changes (e.g., temporal and geographical trends). More attention should be paid to molecular and cellular mechanisms implemented by *L. monocytogenes* to increase its survival; in particular, the role of biofilm should not be underestimated when preventative measures are designed and planned. For instance, in the food industry, the pathogenetic role of this complex structure in the resistance to antimicrobials clearly proven, as well as in the transmission and spread of microbial strains detached from the upper part of the biofilm. In our case, the epidemiological association between isolates found in the refrigerator and detected in clinical specimens might have been underpinned by the formation of biofilm structures, which preserved colonies in stress conditions and favored their persistence [30]. Policies on appropriate cleaning systems should be implemented and scaled-up for processing environmental surfaces and equipment (e.g., bends in pipes, rubber seals, conveyor belts, glasses, etc.), as well as for food [31].

New guidelines and formal rules are needed to improve the management of antimicrobials in different fields, from the food processing chain to animal feeding and human therapies. Global commitment could hinder the emergence and spread of multi-drug resistant microorganisms, including hypervirulent strains of *L. monocytogenes* whose transmission could be fatal in vulnerable population groups.

## Figures and Tables

**Table 1:** Drug susceptibility testing of the human *Listeria monocytogenes* isolate. Clinical and Laboratory Standards Institute (CLSI) 2004 Performance standards for antimicrobial susceptibility testing; 14th informational supplement (Wayne, PA, USA) Vol 24, No. 1, M100-S14.

Antibiotic	Susceptibility	Disc Content (μg)
Ampicillin	Susceptible	10
Chloramphenicol	Susceptible	30
Ciprofloxacin	Susceptible	5
Clindamycin	Resistant	2
Erythromycin	Resistant	15
Gentamicin	Susceptible	10
Imipenem	Susceptible	10
Oxacillin	Resistant	1
Penicillin	Susceptible	10 U
Tetracycline	Susceptible	30
Trimethoprim/Sulfamethoxazole	Resistant	1.25/23.75
Vancomycin	Susceptible	30
